# Optimization of growth regulators on in vitro propagation of *Moringa stenopetala* from shoot explants

**DOI:** 10.1186/s12896-020-00651-w

**Published:** 2020-11-16

**Authors:** Alelegne Yeshamebel Adugna, Tileye Feyissa, Fikresilasie Samuel Tasew

**Affiliations:** 1 Department of Biology, Faculty of Natural and Computational Science, Kotebe Metropolitan University, Addis Ababa, Ethiopia; 2 Institute of Biotechnology, Addis Ababa University, Addis Ababa, Ethiopia; 3 Ethiopian Public Health Institute, Addis Ababa, Ethiopia

**Keywords:** In vitro propagation, *Moringa stenopetala*, Rooting, Shoot multiplication

## Abstract

**Background:**

*Moringa stenopetala* belongs to the flowering family *Moringaceae* and genus *Moringa*. It is often referred to as the East African *Moringa* tree because it is native only to southern Ethiopia and northern Kenya. The expansion of its cultivation and utilization throughout the world especially in Africa is becoming important. For such expansion, the existing propagation method is limiting, so it needs a good propagation system to supply enough planting material with a uniform genotype. Therefore, the main objective of this study was to optimize an in vitro shoot multiplication protocol for *M. stenopetala* by using shoot tip as explants.

**Results:**

Shoots were sterilized and cultured on Muraghige and Skoog (MS) medium for in vitro shoot initiation. For multiple shoot induction, the explants were cultured on MS medium supplemented with different concentrations of kinetin (0.5, 1.0, 1.5, 2.0, 2.5 mg/L) with Indole-3- butyric acid (IBA) or α -naphthalene acetic acid (NAA) (0.01, 0.1, 0.5 mg/L) and maintained at 25 ± 2 °C for four weeks. Rooting was achieved by culturing well developed shoots in half-strength MS medium containing IBA (0.1, 0.5, 1.0, 1.5, 2.0 mg/L), NAA (0.1, 0.5, 1.0, 1.5, 2.0 mg/L), and 0.5 mg/L IBA with NAA (0.1, 0.5, 1.0, 1.5, 2.0 mg/L). Statistical analysis revealed that there was a significant difference among all treatments applied in both shoot multiplication and rooting experiments. The maximum number of shoots per explant (3.43 ± 1.41) and 7.97 ± 4.18 leaves per explant were obtained on MS medium containing 0.5 mg/L kinetin with 0.01 mg/LNAA. The highest mean number of roots per shoot (1.63 ± 1.03) and mean root length (0.87 ± 1.22 cm) were obtained on MS medium containing 1.0 mg/LNAA and 0.1 mg/LIBA alone respectively. After acclimatization, 76% of plants were survived in the greenhouse.

**Conclusion:**

In general, using NAA with kinetin for shoot multiplication was effective than kinetin with IBA. On the other hand, the application of 1.0 mg/L NAA alone and 1.0 mg/L NAA with 0.5 mg/L IBA were more effective for root induction.

## Background

*Moringa stenopetala* “Camel crop”, often referred to as the East African *Moringa,* belongs to the family *Moringaceae* [1], and the genus is represented by 14 species. *M. stenopetala* is a soft drought tolerant fast growing evergreen perennial flowering tree, well adapted to semi-arid areas [2, 3]. Though it grows in many parts of the tropics, it is not as widely known as its close relative, *Moringa oleifera,* but often considered generally more desirable than *M. stenopetala* [4, 5]. In comparison with *M. oleifera*, *M. stenopetala* is more resistant to insect pests [6]. However, a caterpillar of *Noorda trimaculalis* is a known pest for *M. stenopetala*. Mark and Edward et al. [1, 7] stated that the taxonomic position of the family is not clear. It has some features similar to those of *Brassicaceae* and *Capparidaceae* but the seed structure does not agree with either of the above families*.* This indicates that the taxonomic position of the family is not yet settled and is open for further studies. Its seed physiology is also not yet studied in the tropics in general and Ethiopia in particular [8].

In Ethiopia, the tree adapts many ecological regions, including rocky areas along rivers, dry scrubland, and Acacia-Commiphora woodland. It is cultivated in terraced fields, gardens, and small towns [7, 9]. *M. stenopetala* is dominantly found in well-drained soils of southern Ethiopia with an altitudinal range of about 1100–1600 m.a.s.l (meters above sea level) with 500–1400 mm and 24–30 °C annual rainfall and temperature, respectively [10]. Although, the tree has not any specific soil requirements and grow efficiently in neutral soil pH, low temperatures limit the growth of the species in Ethiopia [11].

*M. stenopetala* is propagated both by direct sowing of the seeds without pretreatment and vegetatively using branch cuttings [12]. The optimum temperature for the germination of *M. stenopetala* seeds was reported to be about 25 °C [13]. Optimum light for germination of all *Moringa* species is half shade. When sown in the hotter weather of mid-April, germination percentages for *M. stenopetala* and *M. oleifera* were only 54 and 40%, compared to 92 and 94% in half shade. Seeds should be planted about 2 cm deep in soil that is moist but not too wet [14]. In southern Ethiopian, the ideal season for sowing the seeds from March to August. The time of sowing must be strictly adhered to because the flowering phase should not concede with rainy seasons, which results in heavy flower shedding [15].

*M. stenopetala* is a nutritious crop worldwide and all parts of the tree except the wood are edible. The leaf of *Moringa* is a very popular vegetable in southern Nation Nationalities and the Peoples Regional State of Ethiopia and is valued for its special flavor [2]. The leaf is rich in carbohydrates, proteins, minerals, and essential amino acids. It has more beta carotene than carrots, more protein than peas, more calcium than milk, more potassium than bananas, and more iron than spinach [16].

Besides its food value, many parts of *M. stenopetala* have been used in traditional herbal medicine [17]. Even though the tree has vital value for human and livestock nutrition, its ecology and physiology are remaining uninvestigated [18, 19]. Due to this, *M. stenopetala* is not well known in most parts of the world other than its area of cultivation (Southern Ethiopia and Northern Kenya). Currently, it attracts the attention of scientists across the globe for human health management for its nutritional and medicinal values. Therefore, scientists strive to acquire good propagation systems and strain selection to supply adequate planting materials of superior genotypes.

Therefore, vegetative propagation like cutting is a necessity to obtain uniformity in yield and quality. However, it is less successful due to its slow regeneration and also requires large size cuttings (1–1.5 m long), as well as trees, are grown from cuttings, are known to have much shorter roots or a poor root system [20]. As a result, in vitro propagation methods are the best alternative for the propagation of this plant with uniform genotypes within a relatively short period. Thus, this study was intended to optimize in vitro propagation protocol for *M. stenopetala* so that to evaluate the combined effect of NAA and kinetin and IBA and kinetin on shoot multiplication and to examine the effect of NAA and IBA on rooting of multiplication shoots.

## Results

### Shoot multiplication

The effect of NAA along with kinetin on shoot multiplication after four weeks of culture was highly significant (*P* ≤ 0.05). The results of the present study showed that using NAA in combination with kinetin for shoot multiplication was better than kinetin in combination with IBA.

**Fig. 1:**
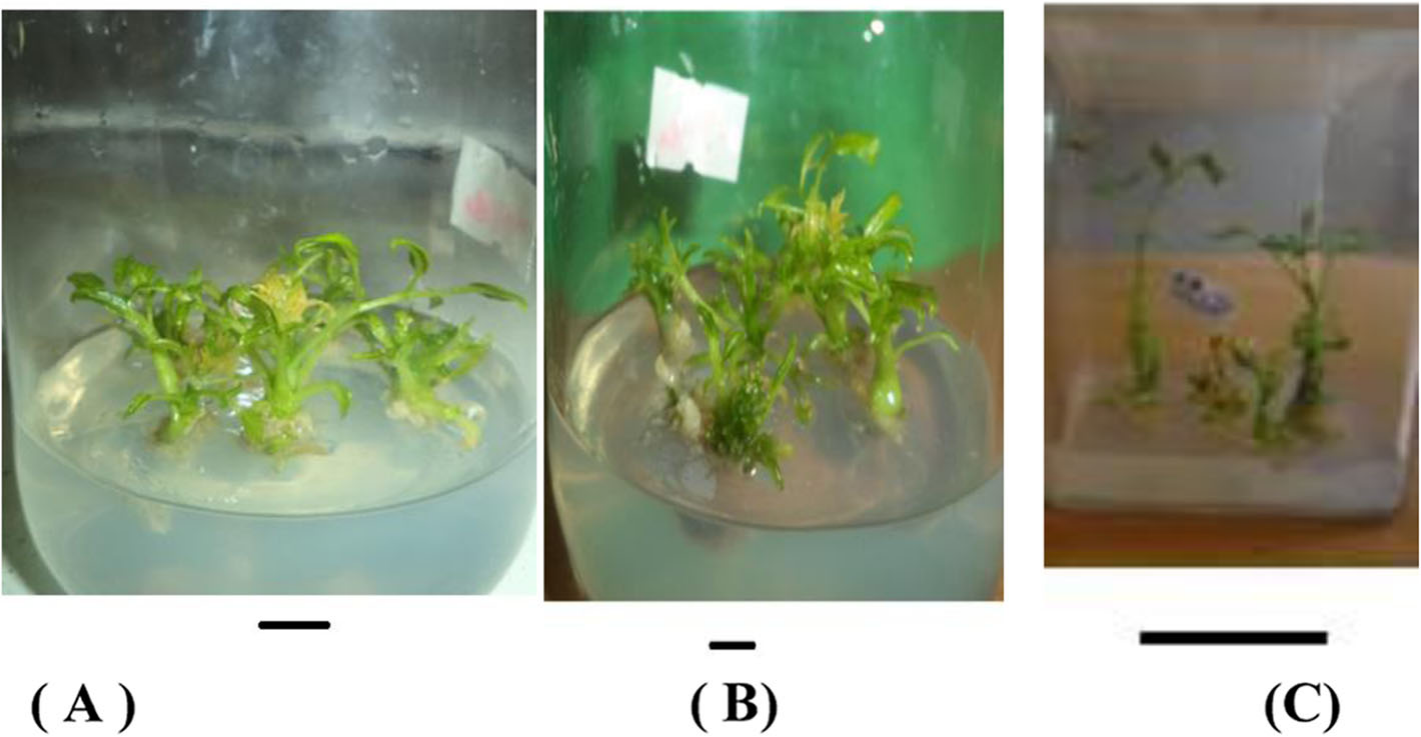
The effect of different growth regulators on shoot multiplication after four weeks of culture; **a** 0.5 mg/l kinetin + 0.01 mg/l NAA, **b** 0.5 mg/l Kinetin + 0.1 mg/l IBA, **c** PGR free. Bar =2 cm

In all treatments, there were differences in the rate of shoot multiplication. The mean number of shoots per explant range from 1.00 ± 0.48 to 3.43 ± 1.41 (Table 1; Table 2; Fig. 1). Among all the treatments, 0.5 mg/L kinetin in combination with 0.01 mg/L NAA resulted in the highest number of shoots per explant (3.43 ± 1.41) (Table 2). The MS medium supplemented with 0.5 mg/L kinetin combined with 0.1 mg/LNAA, 0.5 mg/L kinetin combined with 0.1 mg/L IBA, and 0.5 mg/L kinetin combined with 0.5 mg/L NAA gave the second (2.47 ± 1.36) and the third (2.30 ± 1.90), (2.33 ± 1.85) maximum mean shoot number respectively. Shoot explants cultured on MS medium supplemented with 2.5 mg/L kinetin in combination with 0.5 mg/L NAA and 2.5 mg/L kinetin in combination with 0.5 mg/L IBA produced the lowest mean number of shoots per explant, 1.00 ± 0.48 and 1.00 ± 0.54 respectively. Shoot length was significantly higher on medium without any plant growth regulator (control) as compared to media containing IBA and Kinetin and NAA and kinetin but it produced the lowest mean number of shoots per explant (1.2 ± 1.00). The maximum shoot length (1.10 ± 0.97 cm) and the number of leaves (9.33 ± 10.56) were recorded on MS medium supplemented with 0.5 mg/L Kinetin combined with 0.01 mg/L IBA and 1.0 mg/L kinetin combined with 0.01 mg/L NAA respectively. With increasing the concentrations of IBA and NAA, the number of shoots per explant decreased.

**Table 1:** The effect of different combination of kinetin and IBA on shoot multiplication

KN (mg/l)	IBA (mg/l)	Shoot no. per explant	leaf no. per explant	Shoot length (cm)
0.0	0.0	1.20 ± 1.00^bc^	2.33 ± 1.15^def^	2.67 ± 1.13^a^
0.5	0.01	2.27 ± 1.62^a^	6.60 ± 8.30^bcd^	1.10 ± 0.97^b^
0.5	0.1	2.30 ± 1.90^a^	7.73 ± 7.57^a^	1.08 ± 1.07^b^
0.5	0.5	2.03 ± 1.40^bc^	5.77 ± 6.15^bcde^	0.61 ± 0.31^bc^
1.0	0.01	1.83 ± 1.34^bc^	5.83 ± 7.01^bcde^	1.01 ± 0.72^bc^
1.0	0.1	2.20 ± 1.21^a^	6.77 ± 4.83^bc^	0.90 ± 0.55^de^
1.0	0.5	2.20 ± 1.73^a^	7.40 ± 7.14^a^	0.93 ± 0.98^bc^
1.5	0.01	2.03 ± 1.50^bc^	7.47 ± 6.50^a^	0.99 ± 0.84^bc^
1.5	0.1	1.23 ± .94^bc^	3.83 ± 4.31^bcdef^	0.82 ± 0.67^bc^
1.5	0.5	1.50 ± .94^bc^	2.15 ± 1.45^def^	0.89 ± 0.88^bc^
2.0	0.01	2.20 ± 3.39^a^	2.50 ± 2.08^cdef^	0.66 ± 0.36^bc^
2.0	0.1	1.23 ± 1.14^bc^	2.77 ± 3.90^cdef^	0.60 ± 0.43^bc^
2.0	0.5	1.67 ± 1.30^bc^	3.40 ± 3.77^bcdef^	0.71 ± 0.40^bc^
2.5	0.01	1.13 ± .94^bc^	2.57 ± 2.27^cdef^	0.53 ± 0.40^bc^
2.5	0.1	1.00 ± .64^bc^	1.53 ± 1.78^ef^	0.57 ± 0.46^bc^
2.5	0.5	1.00 ± .54^c^	1.00 ± 1.15^f^	0.44 ± 0.42^c^
Over all means		1.70 ± 1.58^B^	4.49 ± 5.55^A^	0.79 ± 0.70^C^

Numbers within the same column with different letter(s) are significantly different from each other according to Tukey’s multiple range tests at *P* ≤ 0.05. The upper case letters indicate the overall means. The values represent mean ± standard deviation

**Table 2:** Effect of different combination of kinetin and NAA on shoot multiplication

KN (mg/l)	NAA (mg/l)	Shoot no. per explant	Leaf no. per explant	Shoot length (cm) (no. ±SD)
0	0	1.20 ± 1.00^efg^	2.33 ± 1.15^f^	2.67 ± 1.14^a^
0.5	0.01	3.43 ± 1.41^a^	7.97 ± 4.18^bc^	0.68 ± 0.26^b^
0.5	0.1	2.47 ± 1.36^bc^	5.33 ± 3.65^bcde^	0.68 ± 0.24^b^
0.5	0.5	2.33 ± 1.85^cd^	7.20 ± 7.44^bcd^	0.50 ± 0.28^bc^
1.0	0.01	2.20 ± 1.81^cde^	9.33 ± 10.56^a^	0.59 ± 0.39^bc^
1.0	0.1	1.20 ± .96^efg^	3.10 ± 3.28^df^	0.36 ± 0.22^c^
1.0	0.5	1.23 ± 1.04^efg^	3.67 ± 5.06^cde^	0.57 ± 0.40^bc^
1.5	0.01	1.57 ± 1.28^cdefg^	4.23 ± 6.63^cde^	0.46 ± 0.28^bc^
1.5	0.1	1.97 ± 1.50^cdef^	5.10 ± 6.58^bcde^	0.47 ± 0.20^bc^
1.5	0.5	1.43 ± 1.31^defg^	2.23 ± 3.10^f^	0.48 ± 0.40^bc^
2.0	0.01	1.23 ± .73^efg^	1.90 ± 1.03^f^	0.50 ± 0.21^bc^
2.0	0.1	1.10 ± 0.96^fg^	2.40 ± 4.42^f^	0.47 ± 0.28^bc^
2.0	0.5	1.10 ± 0.183^fg^	2.57 ± 2.08^f^	0.53 ± 0.22^bc^
2.5	0.01	1.00 ± 0.31^fg^	1.63 ± 1.03^f^	0.55 ± 0.28^bc^
2.5	0.1	1.00 ± 0.38^fg^	1.53 ± .94^f^	0.47 ± 0.27^bc^
2.5	0.5	1.00 ± 0.48^fg^	1.23 ± 1.19^c^	0.36 ± 0.29^c^
Overall means		1.57 ± 1.36^B^	3.96 ± 5.40^A^	0.51 ± 0.30^C^

Numbers within the same column with different letter(s) are significantly different from each other according to Tukey’s multiple range tests at *P* ≤ 0.05. The upper case letters indicate the overall means. The values represent mean ± standard deviation

### Rooting

**Fig. 2:**
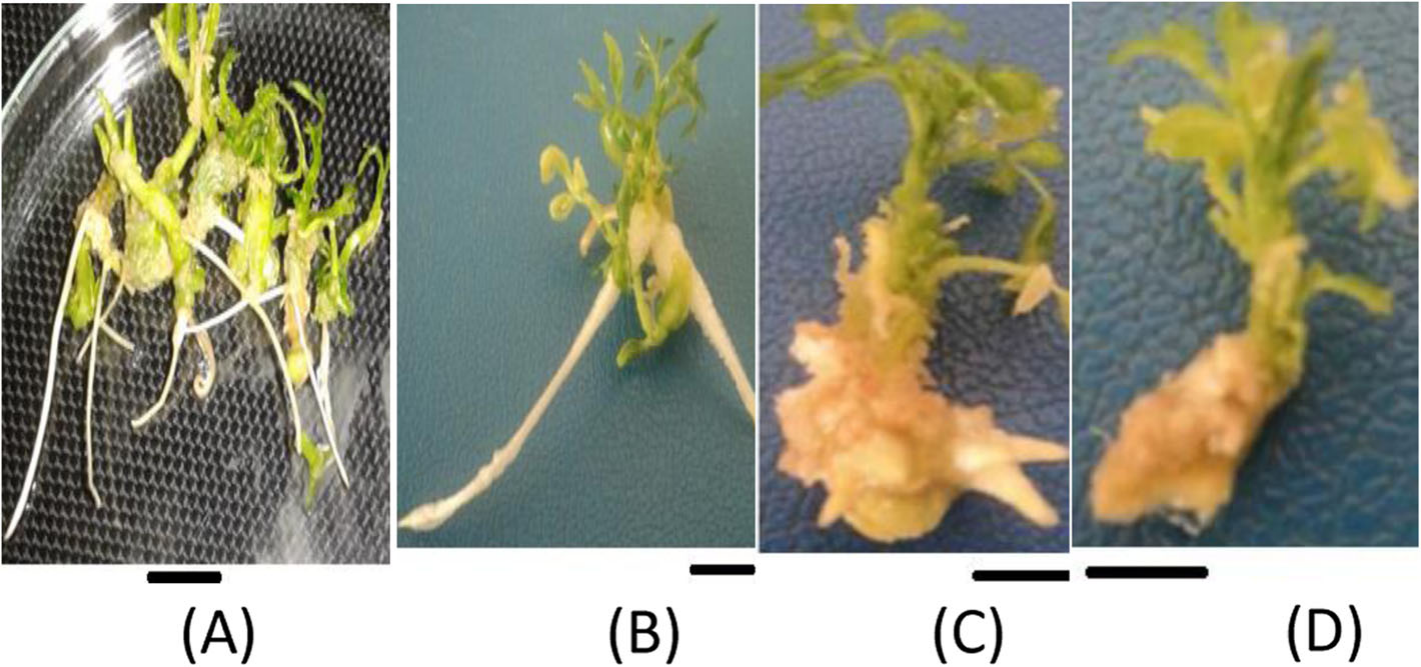
The effect of NAA and IBA on rooting of micro shoots after four weeks of culture. **a** 1.0 mg/l NAA, **b** 1.0 mg/l IBA, **c** 1.0 mg/l NAA in combination with 0.5 mg/l IBA, **d** PGR free medium. Bar = 2 cm

The application of NAA alone exhibited the maximum mean root number per shoot as compared to IBA alone and IBA in combination with NAA. The highest mean root number per shoot (1.63 ± 1.03) and mean root length (0.87 ± 1.22 cm) were obtained on medium containing 1.0 mg/L NAA and 0.1 mg/L IBA, respectively (Table 3). The second (1.50 ± 0.38) and third (1.23 ± 0.70) highest mean root number was obtained on a medium containing 1.0 mg/L IBA alone and 0.5 mg/LIBA respectively and the second (0.84 ± 0.54 cm) the third (0.67 ± 0.28 cm) highest mean root length were obtained on medium containing 1.0 mg/L NAA and IBA alone respectively. The lowest mean number of roots per explant was produced on growth regulator free medium (0.26 ± 0.57), 2.0 mg/L NAA (0.27 ± 0.45), and 2.0 mg/L NAA along with 0.5 mg/L IBA (0.27 ± 0.58). Moreover, the effect of NAA and IBA on the rooting of micro shoots after four weeks of culture is shown in Fig. 2.

**Table 3:** Effect of NAA and IBA on rooting of micro shoots

NAA (mg/l)	IBA (mg/l)	Root no. per explant	Root length (cm)
0	0	0.26 ± 0.57^bc^	0.12 ± 0.21^bc^
0	0.1	1.03 ± 1.47^c^	0.87 ± 1.22^a^
0	0.5	1.23 ± 0.70^b^	0.43 ± 0.43^b^
0	1.0	1.50 ± 0.38^a^	0.67 ± 0.28^c^
0	1.5	0.13 ± 0.35^bc^	0.15 ± 0.48^bc^
0	2.0	0.07 ± 0.25^bc^	0.05 ± 0.20^bc^
0.1	0	1.07 ± 1.86^bcd^	0.34 ± 0.55^cd^
0.5	0	1.23 ± 1.19^bc^	0.52 ± 0.51^bc^
1.0	0	1.63 ± 1.03^a^	0.84 ± 0.54^a^
1.5	0	0.67 ± 0.84^cd^	0.23 ± 0.28^cd^
2.0	0	0.27 ± 0.45^d^	0.14 ± 0.24^d^
0.1	0.5	0.50 ± 0.97^bc^	0.13 ± 0 .21^c^
0.5	0.5	0.67 ± 1.16^bc^	0.24 ± 0.37^bc^
1.0	0.5	1.20 ± 1.16^a^	0.43 ± 0.40^a^
1.5	0.5	1.03 ± 1.13^a^	0.41 ± 0.46^a^
2.0	0.5	0.27 ± 0.58^f^	0.09 ± 0 .19^c^

Numbers within the same column with different letter(s) are significantly different from each other according to Tukey’s multiple range tests at *P* ≤ 0.05. The upper case letters indicate the overall means. The values represent mean ± standard deviation

### Acclimatization

**Fig. 3:**
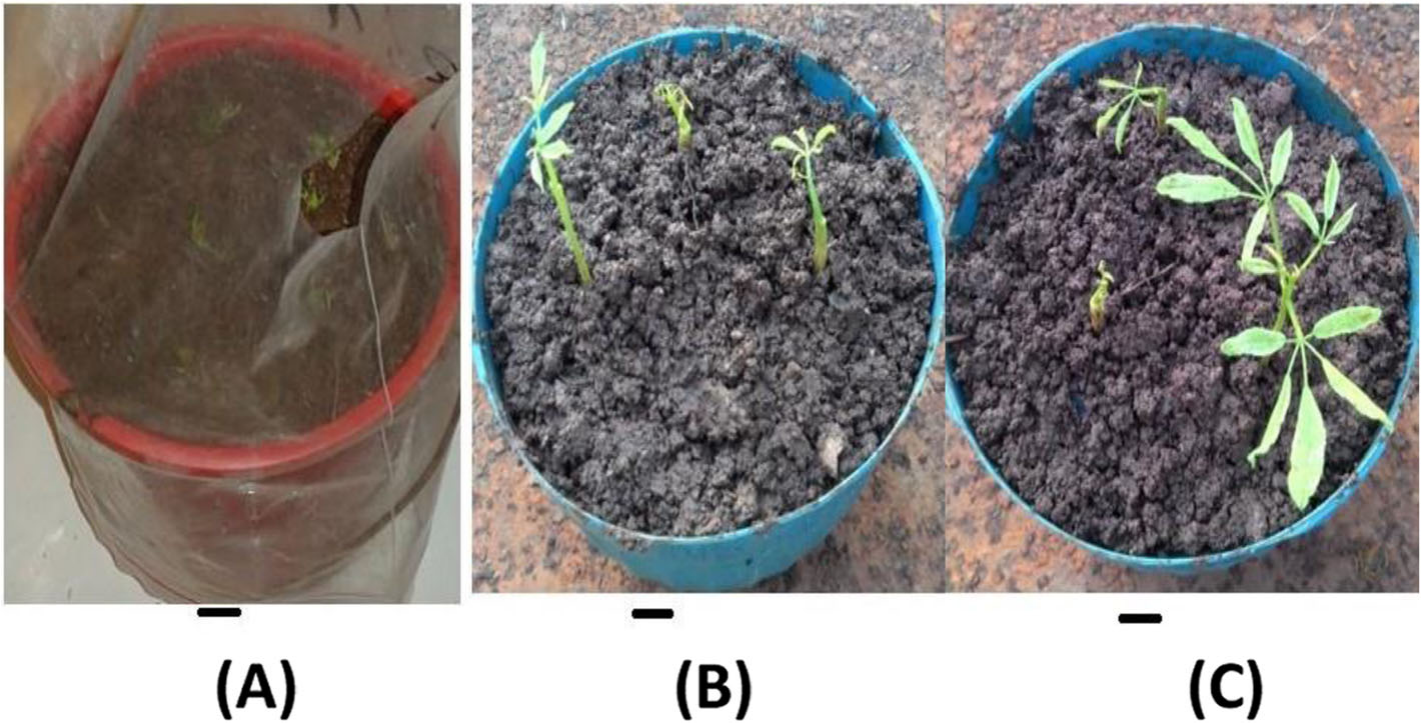
Acclimatization; **a** During transplanting, **b** After three weeks, **c** After four weeks. Bar = 2 cm

Among 50 plantlets acclimatized in the greenhouse, 38 (76%) plantlets were survived (Fig. 3).

## Discussion

### Effect of kinetin, IBA, and NAA on shoot multiplication

In the present study, efforts have been made to optimize in vitro propagation protocol for *M. stenopetala*. Surface sterilization of seeds with 10% NaOCl solution for 25 min was effective to reduce microbial contaminants. In line with this finding, sterilization of *Moringa concanensis* seeds was effective for 15 min in 10% NaOCl solution [21]. The disparity in time duration it might be due to the nature of seeds in these two different plant species. In this study, there was 100% initiation of the shoot after four weeks of the culture of the shoot tip in shoot initiation media supplemented with BAP alone. This is similar to the finding of [22] who got 100% initiation of shoots from stem segment explants taken from 10-day old seedlings of *M. oleifera*.

The present study also revealed that the effect of cytokinin in combination with auxin was compared. Thus, the application of 0.5 mg/L kinetin along with 0.01 mg/L NAA resulted in the maximum mean numbers of shoots (3.43 ± 1.41) with 7.97 ± 4.18 mean number of leaf and 0.67 ± 0.26 cm means shoot length per explant. Besides, there was a continuous decrease in the number of shoots when the concentration of NAA increased from 0.01 to 0.1 mg/L combined with kinetin. Moreover, a high cytokinin to auxin ratio favors shoot formation.

The induction of multiple shoots in *M. concanensis* has been previously characterized by different growth regulators. Fatima et al. [21] on nodal explants of *M. concanensis* obtained 11.00 ± 1.15 mean shoot number with 5.00 ± 1.95 cm mean length on MS medium supplemented with 0.1 mg/L kinetin along with 0.05 mg/L NAA. However, in this study, the maximum mean number of shoots per explant (3.43 ± 1.41) with a mean shoot length of 0.68 ± 0.26 cm was recorded at the concentration of 0.5 mg/L kinetin in along with 0.01 mg/L NAA. Even though the result reported by these authors is greater than the present study, the trend was similar. In both studies relatively at low concentration of NAA and high concentration of kinetin conforms to the best result. The probable difference is due to the genotype difference, source of explants, and the difference in concentration of kinetin and NAA. In addition, the performance of kinetin alone in shoot length is further supported by the sister family (*Brassicaceae)* of *Moringa*. The study in *Matthiolaincana (Brassicaceae*) showed that MS medium supplemented with 2.0 mg/L kinetin without NAA resulted in the best shoot length [23]. In the present study, the medium containing 2.0 mg/L kinetin along with 0.01 NAA produced 1.23 ± 0.73 mean number of the shoot with 0.50 ± 0.21 cm means shoot length. This implies that the combined effect of auxin may inhibit the effectiveness of cytokinin in shoot development. In addition, the difference in genotype constituent and source of explant might result in a difference in the aforementioned studies.

In the present study, a combination effect of kinetin and IBA was also evaluated on shoot multiplication. Thus, shoot explants cultured on MS medium supplemented with 0.5 mg/L kinetin with 0.1 mg/L IBA exhibited both the maximum mean number shoots and leaves of 2.30 ± 1.90 and 7.73 ± 7.57, respectively per explant. The result from the combined effect of IBA and kinetin was found to vary with the concentration of both hormones in the mean number of shoot, leaf, and shoot length. When the concentration of kinetin was greater than that of IBA, relatively better result was recorded. This is due to the effect of cytokinin as it promotes the axilliary branching or axilliary bud proliferation [24]. Although both auxin and cytokinin are usually required for growth or morphogenesis, auxin inhibits cytokinin accumulation [25] while cytokinin can inhibit at least some of the actions of auxin. There was a continuous decrease in the number of shoots when the concentration of IBA increased from 0.01 to 0.5 mg/L combined with kinetin. However, the combined effect of kinetin and NAA exhibited more effective as compared to the combined effect of IBA and kinetin [25, 26].

Alkhateeb [26] reported that *M. peregrina* (Forsk) tested using MS medium supplemented with 1.0 mg/L kinetin resulted in a higher mean shoot number. On the other hand, the above-mentioned author reported a significantly reduced number of leaves in micro-shoot using relatively high levels of kinetin. In this study, when the concentration of kin raised from 0.5 to 2.5 mg/L along with increasing IBA concentration from 0.01 mg/L to 0.5 mg/L lower number of shoot per explant (1.0 ± 0.54) with 0.44 ± 0.42 cm shoot length was obtained for *M. stenopetala*. In contrast to [26], the present study revealed that growth regulators free MS medium resulted in an elongated shoot length compared to MS media supplemented with growth regulator hormone. In the present study, the MS medium supplemented with 0.5 mg/L kin along with 0.1 mg/L IBA resulted in the maximum number of the shoot (2.30 ± 1.90), and the mean number of leaves (7.73 ± 7.57). This finding is in agreement with the finding of [27], who got a simultaneous increase number of nodes from 3.61 to 4.64 when the concentration of kinetin increased from 0.5 to 2.0 mg/l in *Matthiola incana*. From the aforementioned statements, type and concentration of growth regulators and species genotype seem the most important factors in in vitro shoot multiplication.

### The effect of NAA and IBA on rooting

The analysis of variance revealed that the root number and root length varied significantly with half-strength MS medium supplemented with NAA, IBA, and the combination of both. The application of NAA alone exhibited the maximum mean root number per shoot as compared to IBA alone and IBA with NAA. The highest number of mean roots per shoot (1.63 ± 1.03) and mean root length (0.87 ± 1.22 cm) were obtained at 1.0 mg/L NAA and 0.1 mg/L IBA alone, respectively.

Furthermore, increasing NAA from 0.0 to 1.0 mg/L at 0.0 mg/l IBA concentration showed a significant increase in the mean number of roots per shoot from 0.26 ± 0.57 to 1.63 ± 1.03, and mean root length from 0.12 ± 0.21 to 0.84 ± 0.54 cm. However, a further increase in the concentration of NAA from 1.0 to 2.0 mg/L showed a reduction in the mean root number per shoot and mean root length from 1.63 ± 1.03 to 0.27 ± 0.45 and 0.84 ± 0.54 to 0.14 ± 0.24 cm, respectively. The same trend was observed in both treatments: concentration of IBA alone and with NAA. Increasing IBA from 0.0 to 1.0 mg/L increased both the number of roots per shoot from 0.26 ± 0.57 to 1.50 ± 0.38 and mean root length from 0.12 ± 0.21 to 0.67 ± 0.28 cm. Further increase in the concentration of IBA from 1.0 mg/L to 2.0 mg/L, mean root number was reduced from1.50 ± 0.38 to 0.07 ± 0.25 and mean root length from 0.67 ± 0.28 to 0.05 ± 0.20 cm, respectively. In agreement with the present finding, [28] reported the decreasing number of the root when the concentration of IBA is greater than 2.0 mg/L. Moreover, the above-mentioned authors indicated that the inhibitory effect of a high concentration of auxin on root formation is caused for such decreasing. In addition to concentration variations, the difference in the genotype of the explant can determine root formation. Therefore, based on the present finding, the optimum concentration is between1.0 and 2.0 mg/L for root formation in *M. stenopetala*.

The combined effect of NAA and IBA on root induction confirmed a medium effect than the effect rendered by each hormone. Increasing NAA from 0.1 to 1.0 mg/L at 0.5 mg/L IBA showed a significant increase from 0.50 ± 0.97 to 1.20 ± 1.16 in mean number of roots per shoot and from 0.13 ± 0.21 cm to 0.43 ± 0.40 cm mean root length. However, a further increase in the concentration of NAA above 1.0 mg/L by keeping IBA concentration at 0.5 mg/L reduced the mean root number from 1.20 ± 1.16 to 0.27 ± 0.58.

In the combination of 1.0 mg/L NAA with 0.5 mg/L IBA, the mean number of roots and mean root length was 1.20 ± 1.16 and 0.43 ± 0.40 cm, respectively. On the other hand, the number of roots and mean root length was 0.27 ± 0.58 and 0.09 ± 0.19 cm, respectively at 2.0 mg/L NAA and 0.5 mg/L IBA.

Stephenson and Fahey [29] obtained 4.7 roots per explant in 1/2 strength MS medium supplemented with 0.5 mg/L NAA in *M. oleifera*. In contrast, Islam et al. [22] reported that the hormone-free medium was ideal for rooting. In the present study, less mean root numbers (1.23 ± 1.19) was obtained at 0.5 mg/L concentration and growth regulator free medium (0.26 ± 0.57) as compared to the result reported by the aforementioned authors. In the present study, the highest mean number of root and mean root length per explant was 1.63 ± 1.03 and 0.87 ± 1.22 cm in MS medium containing 1.0 mg/L NAA and 0.1 mg/L IBA, respectively. Islam et al. [22] stated that medium supplemented with 1.0 and 2.0 mg/L NAA was not inducing rooting at all. In addition, these authors also stated that the medium supplemented with 0.05, 1.0 and 2.0 mg/L IBA produced 0.0, 2.0, and 2.8 mean numbers of roots per explant respectively. In this study, the first, second, and least mean root numbers were produced at concentrations of 1.0 mg/L NAA, 1.0 mg/L IBA, and 2.0 mg/L IBA, respectively. When the concentrations of IBA increased from 0.05 to 2.0 mg/L, the mean number of roots per explant also increased. The trend reported by [22] agreed with the present study particularly in the effect of IBA. The result of the present study indicated that when the concentration of IBA increased from 0.1 to 1.0 mg/L, the mean number of roots per explant continuously increased but further increasing of IBA concentration beyond 1.0 mg/L led to a decreasing mean number of roots per explant. In contrast with the present finding, Islam et al. [22] found the highest number of roots per explant at 2.0 mg/L IBA.

In agreement with the finding of [26] on *M. peregrina* (Forsk) who reported the maximum number of roots in MS medium supplemented with 1.0 mg/L IBA and MS medium containing 0.5 mg/L IBA produced longer roots than control or medium supplemented with different levels of NAA. Here, the medium supplemented with1.0 mg/L IBA alone showed the highest rate of root induction (1.50 ± 0.38) with longer roots at 0.1 mg/L IBA (0.87 ± 1.22) compared to the control medium. As results showed, using lower levels of auxin (NAA or IBA) favors higher levels of root induction and elongation. This implies that a higher concentration of growth regulators inhibits root elongation [30]. This is might be related to ethylene deposition and poor vascular connection of the root with the stem because of callus intervention [30].

In an experiment for in vitro rooting of *M. oleifera*, when micro-shoots were cultured on a medium containing 0.5 mg/L IAA with IBA at 1.0 mg/L resulted in the highest number of root induction [31]. These variable responses could be due to genetic differences, differences in the explant source, the concentration difference of growth regulators, and the type and/or age of explants used to establish the cultures [32].

### Acclimatization

About 76% of acclimatized plantlets were survived in the greenhouse. This survival rate is similar to the result of [31, 32] on *M. oleifera*. In a shaded greenhouse experiment, 80% of *M. oleifera* was survived [32]. Saini et al. [31] also obtained the same result as [28], provided that the potted plantlets were covered with clear polythene bags and kept in a shaded greenhouse for 15 days before exposure to ambient conditions.

## Conclusions

Large-scale propagation of *M. stenopetala* by tissue culture methods is feasible and several plantlets can be regenerated from a single shoot tip explant. Using NAA with kinetin for shoot multiplication was effective than kinetin with IBA. Therefore, 0.5 mg/L kinetin combined with 0.01 mg/L NAA was found to be optimal in producing the maximum number of shoots per explant. Application of NAA at a concentration of 1.0 mg/L and 1 mg/L NAA combined with 0.5 mg/L IBA was more effective for root induction. This implies that this protocol enables the mass propagation of *M. stenopetala* from shoot tip explant. Therefore, further optimization of this protocol may be required for the mass propagation of this plant. Further optimization of this protocol may also require low-cost mass propagation of this plant by reducing the media components used in this protocol or substituting with cheaper components and methods. It is also recommended to practice other techniques like culturing of explants in liquid medium reduces the cost of agar for mass propagation of *M. stenopetala.*

## Methods

### Source of explant and surface disinfection

Matured seeds of *M. stenopetala* were collected from Merab Abaya, Arba Minch area, in the Southern Nation Nationalities and the Peoples Regional State of Ethiopia during November and December of 2015. We got permission to collect the sample from local authorities and oral consent from local societies after we did a discussion about the benefit of the research. The collected specimens were identified and confirmed by the botanist before we did the further experimental analysis. Fruits were cut open and the seeds were separated from pods and washed with local detergent (omo) for 10 min and rinsed in running tap water for five minutes then thoroughly washed and rinsed again in sterilized distilled water for about 24 h to speed up seed germination.

Seeds were surface sterilized with 10% NaOCl solution (sodium hypochlorite solution) for 25 min followed by five washing with sterile distilled water and sown in culture jars containing 50 ml plant growth regulators-free MS [33] medium under aseptic conditions. The MS medium was enriched with 30 g/l sucrose (w/v), 8 g/L agar (w/v), pH was adjusted to 5.8, and autoclaved at 121 °C with a pressure of 105 KPa for 15 min. The cultures were maintained in a growth room under a light intensity of 16-h photoperiod provided by cool-white fluorescent lamps at a temperature of 25 ± 2 °C.

### Source of reagents/micro and macronutrients/

Macronutrients, micronutrients, vitamins, and hormones that have been used in this research were manufactured in different com panies. Particularly, most of these are manufactured from Germany but some of them are from the USA and Japan.

Macronutrients (NH4NO3, KNO3, CaCl2.2H2O, KH2PO4) were purchased from Germany but MgSO4.7H2 was from Japan), and micronutrients (ZnSO4.7H2O, H_3_BO_3_, MnSO4.4H2O, CuSO4.5H2O, Na2MoO4.2H2O, KI, COCl2.6H2O were purchased from Germany but Fe-Na-EDTA was purchased from the USA). Besides organic supplements (Myo-inositol, Glycin**e,** Nicotinic acid, Pyridoxin (B6), Thiamin (B1), and hormones (NAA, IBA, and kinetin were purchased from Germany in Sigma-Aldrich company.

### Preparation of stock solution and culture media

#### MS stock solution preparation

The stock solution of macronutrients, micronutrients, and vitamins was prepared separately by weighing the recommended amount of powder by dissolving in distilled water and stored them in a refrigerator at a temperature of + 4 °C until used. The prepared stock solutions were stored at + 4 °C for a maximum of one month.

#### Growth regulators stock solution preparation

In this study, different plant growth regulators namely cytokinins (kinetin) and two auxins (IBA, NAA) were used with different concentrations and combinations. Each of these growth regulator stock solutions was prepared by weighing and dissolving the powder in distilled water at a concentration of 1.0 mg/ml. To begin the dissolving process 3–4 drops of 1 M NaOH or 1 M HCl were added based on the requirement of the growth regulators (NaOH for auxin, HCl for cytokinin). Then, the volume was adjusted by adding distilled water. Finally, growth regulators stock solutions were stored in a refrigerator at a temperature of + 4 °C until used.

#### Culture media preparation

Culture media were prepared by mixing the proper amount of MS stock solutions (50 ml/L macro, 5 ml/L micro, 5 ml/L iron EDTA and 5 ml/L vitamin) then 30 g/L sucrose was added to the solution as energy and carbon source. After mixing all the components and adjusting the volume, growth regulators were added as required and pH was adjusted to 5.8 using 1 N NaOH or 1 N HCl. Finally, 8.0 g/L agar was added. After melting the agar by using a hot plate, a 50-ml medium was dispensed into each Magenta GA-7 culture vessel. The medium was sterilized by autoclaving at a temperature of 121 °C at a pressure of 105 KPa for 15 min. Finally, the medium could cool in the laminar airflow cabinet.

The media compositions that were used for different experiments were as follows:

##### Media for shoot multiplication

- *MS + IBA and Kinetin + 30 g/L sucrose + 8 g/L agar*
- *MS + Kinetin + NAA + 30 g/L sucrose + 8 g/L agar*

##### Media for Rooting

- *1/2 MS + IBA + 30 g/L sucrose + 4 g/L agar*
- *1/2MS + NAA + 30 g/L sucrose + 4 g/L agar*
- *1/2MS + IBA+ NAA + 30 g/L sucrose + 4 g/L agar*

### Culture conditions

All types of cultures were kept in a growth room at a temperature of 25 ± 2 °C and under a light intensity of 16-h photoperiod provided by cool-white fluorescent lamps.

### Shoot initiation

Shoot tips obtained from in vitro germinated seedlings were used for culture initiation. Shoot tips from in vitro germinated seedlings were excised and cultured on MS medium supplemented with BAP (0.5, 1.0, 1.5, 2.0, 2.5 mg/L) alone and in combination with NAA (0.1, 0.5 and 1.0 mg/L). The medium was supplemented with 30 g/L sucrose (w/v) and the pH was adjusted to 5.8 before the addition of 8 g/L agar (w/v). Six shoots per Magenta culture vessel was considered as a unit of replication and there were five replications for each treatment. The cultures were maintained in a culture room under a light intensity of 16-h photoperiod provided by cool-white fluorescent lamps at a temperature of 25 ± 2 °C.

### Shoot multiplication

The in vitro initiated shoots were cultured on MS medium supplemented with different concentrations of kinetin (0.0, 0.5, 1.0, 1.5, 2.0, 2.5 mg/L) in combination with IBA (0.0, 0.01, 0.1, 0.5) or kinetin (0.0, 0.5, 1.0, 1.5, 2.0, 2.5 mg/L) in combination with NAA (0.01, 0.1, 0.5 mg/L) to determine their effect on multiple axillary shoot formation. Growth regulators, free MS basal medium were used as a control. Six shoots per Magenta culture vessel was considered as a unit of replication and there were five replications for each treatment. The cultures were maintained at 25 °C ± 2 °C with 16 h photoperiod and sub-cultured every four weeks. Shoot length, the number of leaves, and the number of shoots per explant were recorded after four weeks.

### Rooting

Micro shoots obtained from the shoot multiplication medium were transferred to the rooting medium. The rooting medium was half-strength MS medium containing different concentrations of NAA and IBA. Six shoots were cultured in each culture vessel and a total of five replications were designed for each treatment. The cultures were maintained in the growth room under the same condition as of shoot multiplication. The number of roots per shoot and root length was recorded after 4 weeks.

### Acclimatization

Plantlets with well-developed shoots and roots were taken out of the culture jars and the roots were washed thoroughly with running tap water and transferred into pots containing autoclaved (sterilized) sand, red soil, and compost at a ratio of 2:1:1 respectively. The plantlets were covered with a transparent plastic bag to maintain moisture for two weeks and placed them in the shaded region of the tissue culture room prior to their transfer to the greenhouse condition and watered within two days interval (morning and evening). The plastic cover was gradually removed after one month and the plantlets were successfully established in the greenhouse.

### Data analyses

After four weeks of transferring explants into multiplication media, the number of shoots per explant, mean shoot length, and the number of leaves were recorded. After four weeks of transferring well-developed shoots into rooting media, the number of roots per shoot, and mean root length were recorded. All data were subjected to analysis of variance (ANOVA) to quantify the differences between applied treatments. Treatment means were separated using the least significant differences (LSD) at a probability level of *p* ≤ 0.05. ANOVA table was constructed using SPSS computer software of version 20 and Tukey’s multiple range tests were used.

## Data Availability

The datasets used and/or analyzed during the current study available from the corresponding author on reasonable request.

## Abbreviations

2, 4-D: 2, 4-Dichlorophenoxyacetic acid
2-ip: Isopentenyl-adenine
BAP: 6- Benzylamino purine
IAA: Indole acetic acid
IBA: Indole-3- butyric acid
Kin: Kinetin
MS: Murashige and Skoog
NAA: α -naphthalene acetic acid
PGR: Plant growth regulator
TDZ: Thidiazuron
CRD: Complete Random Design
ANOVA: Analysis of Variance
SPSS: Statistical Package for Social Science
LSD: Least Significance Difference
